# A comparison of diagnostic accuracy between CNN and transformer AI models for periodontal bone loss detection: A systematic review and meta-analysis

**DOI:** 10.34172/japid.4275

**Published:** 2026-09-12

**Authors:** Shaula Nada Aulia, Fabillah Haikal Azizi, Muhammad Hafizh Ash-Shiddiq, Amy Nindia Carabelly, Selviana Rizky Pramitha

**Affiliations:** ^1^Undergraduate Program in Dentistry, Faculty of Dentistry, Lambung Mangkurat University, Banjarmasin, Indonesia; ^2^Department of Oral and Maxillofacial Pathology, Faculty of Dentistry, Lambung Mangkurat University, Banjarmasin, Indonesia; ^3^Department of Oral Medicine, Faculty of Dentistry, Lambung Mangkurat University, Banjarmasin, Indonesia

**Keywords:** Artificial intelligence, Computer vision, Convolutional neural networks, Diagnostics, Periodontal bone loss

## Abstract

**Introduction:**

Convolutional neural networks (CNNs) and transformers are artificial intelligence (AI) models used for accurate periodontal bone loss (PBL) diagnosis. This study compared the diagnostic accuracy between CNN and transformer models in detecting PBL.

**Methods:**

We searched ten databases: PubMed, Scopus, Cochrane, Embase, WorldCat, Lens, Google Scholar, Taylor & Francis, ScienceDirect, and ClinicalTrials.gov from 2015 to 2025 to identify studies that used CNN and/or transformer models on human dental radiographs, evaluating diagnostic performance based on area under the curve, accuracy, sensitivity, specificity, correlation matrix, F1-score, precision, and recall. Risk of bias was assessed using PROBAST+AI. Meta-analyses using random-effects models estimated pooled diagnostic odds ratio (DOR), sensitivity, and specificity.

**Results:**

Seventeen studies met the eligibility criteria: 14 evaluated CNN-based models, and 3 evaluated transformer-based models, comprising a total of 26,502 cases and 55,076 controls. The overall risk of bias was low. CNN models demonstrated pooled sensitivity and specificity of 0.85 (95% CI: 0.77–0.90) and 0.88 (95% CI: 0.80–0.93), with a DOR of 41.69 (95% CI: 19.41–89.52). Transformer models showed a sensitivity of 0.87 (95% CI: 0.74–0.94), specificity of 0.80 (95% CI: 0.34–0.97), and DOR of 25.64 (95% CI: 3.47–189.63). Heterogeneity was substantial (I^2^>98%), with radiograph type and study design identified as significant contributors. CNN results remained robust across sensitivity analyses, while transformer estimates were more sensitive to individual studies.

**Conclusion:**

Current evidence demonstrates greater diagnostic accuracy of CNN-based models for periodontal bone loss detection, while comparative conclusions for transformer-based models remain premature.

## Graphical Abstract

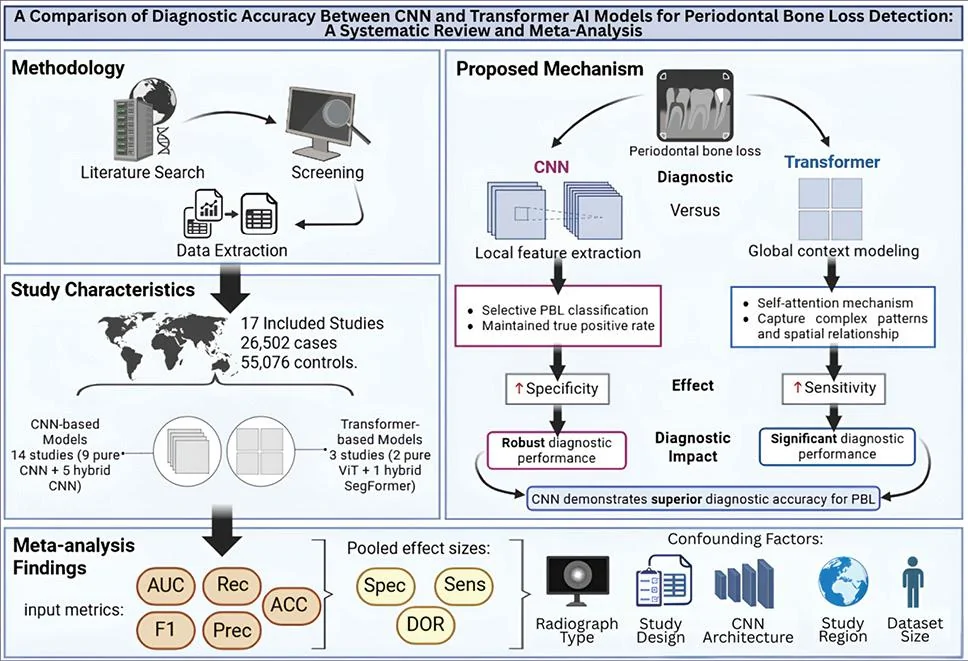

## Introduction

 Periodontitis is an inflammatory disease involving progressive damage to the alveolar bone, gums, and periodontal ligaments surrounding the teeth.^1^ With a prevalence rate of 74.1% in Indonesia, periodontitis can lead to periodontal bone loss (PBL) and become a risk factor for systemic diseases.^1–3^ PBL represents the intersection between inflammation caused by disruption of the oral microbiota and potential systemic complications, making PBL an indicator for the diagnosis of periodontitis and screening for systemic disease risk.^4^ Accurate PBL detection is vital for effective treatment of periodontitis, but conventional clinical exams and manual radiograph interpretation lack sensitivity for early bone loss and are prone to variability.^2,5,6^ Current trends indicate an increase in the adoption of artificial intelligence, especially convolutional neural networks (CNNs) and transformers, in the automatic assessment of PBL severity.^7,8^ CNNs have long dominated medical image analysis by extracting spatial features from radiographs, whereas transformers, particularly vision transformers (ViT), model images as patch sequences to capture complex anatomical relationships.^7,9^ Both architectures enable automated, consistent, and quantitative assessment of PBL, thereby improving diagnostic accuracy and supporting more targeted periodontitis therapy.^1^

 Zhang et al.^7^ (2025) reported that ViT models achieved 92% accuracy, an F1-score of 0.95, and an AUC of 98% in classifying furcation involvement on panoramic radiographs, outperforming conventional CNN. ViT offered better interpretability through attention mechanisms and was particularly effective in capturing global contextual features in wide-field images.^7^ CNNs, however, have consistently shown strong and reliable performance across studies, especially in binary classification tasks and on high-resolution, focused images such as periapical radiographs.^2^ DenseNet121 with GAP and mRMR by Guler Ayyildiz et al.^10^ (2024) achieved 79.5% accuracy and an AUC of ~0.77, while a custom CNN by Krois et al.^11^ (2019) reached 81% accuracy, comparable to experienced clinicians.^10,11^ CNNs are also more computationally efficient and require less data, making them suitable for smaller datasets or resource-limited settings, but their performance may decline in complex image environments such as panoramic scans where overlapping anatomy obscures features.^12^ In contrast, ViTs have shown greater robustness in such settings, with Yu et al.^13^ (2024) and Zhang et al.^7^ (2025) reporting high agreement with expert assessments in full-arch radiographic analyses.^7,13^

 These findings highlight the importance of systematically evaluating the relative performance of CNNs and transformers in PBL detection. Although CNNs have long dominated PBL detection, the emergence of transformers offers flexibility and superior performance in capturing global spatial dependencies. Given the fundamental differences between the architectures, it is important to evaluate whether transformers can provide higher diagnostic accuracy than CNN in detecting PBL. To date, no systematic review has compared the diagnostic accuracy of the two architectures. Therefore, this study aimed to compare the diagnostic accuracy between CNN and transformer models in detecting PBL.

## Methods

###  Protocol and Registration

 This systematic review is in the process of being prospectively registered in PROSPERO (CRD420251250804). Our study adheres to the relevant components of the Preferred Reporting Items for a Systematic Review and Meta-analysis (PRISMA) 2020 guidelines for Diagnostic Test Accuracy studies.^14^ All stages of the review (title and abstract screening, full-text screening, data extraction, and risk of bias assessment) were conducted in duplicate by two independent reviewers (SNA and MHA), with any disagreements resolved through discussion with a third independent reviewer (FHA).

###  Research Question

 The research question was structured using the Population–Intervention–Comparator–Outcome (PICO) framework. The population (P) comprised adults undergoing anterior and/or posterior dental radiographs showing periodontal condition, either normal or with periodontal bone loss. The intervention (I) was the use of deep learning models based on transformer architectures for automated detection of periodontal bone loss, while the comparator (C) was the use of deep learning models based on CNNs for the same purpose. The outcomes (O) were diagnostic performance metrics, including area under the receiver operating characteristic curve (AUC ROC), overall diagnostic accuracy (ACC), sensitivity (Sens), specificity (Spec), correlation metrics such as Pearson’s correlation coefficient (PCC) and intraclass correlation coefficient (ICC), as well as F1-score (F1), precision (Prec), and recall (Rec).

###  Search Strategy

 We performed a comprehensive literature search across 10 electronic databases: PubMed, Scopus, Cochrane, Embase, WorldCat, Lens, Google Scholar, Taylor & Francis, ScienceDirect, and ClinicalTrials.gov, covering the period from January 2015 to June 2025. Only original research articles published in English were considered. Review articles, protocols, conference abstracts, editorials, and case reports were excluded. Table 1 presents the complete search strategies, including keyword combinations and the number of articles retrieved from each database.

**Table 1:** Databases and research method

**Database**	**Keyword**	**Hits**
PubMed	("periodontal bone loss" OR "alveolar bone loss") AND ("convolutional neural network" OR "CNN" OR "transformer" OR "vision transformer" OR "ViT" OR "Swin Transformer")	21
Scopus	("periodontal bone loss" OR "alveolar bone loss") AND ("deep learning" OR "artificial intelligence" OR "machine learning") AND ("convolutional neural network" OR "CNN" OR "transformer" OR "vision transformer" OR "ViT" OR "Swin Transformer")	49
Cochrane	ID Search (Hits) #1 "periodontal bone loss" (20) #2 "alveolar bone loss" (1967) #3 "deep learning" (1321) #4 "Artificial intelligence" (3130) #5 "machine learning" (3485) #6 "CNN" (328) #7 "Convolutional neural network" (535) #8 "Transformer" (77) #9 "Vision transformer" (3) #10 "ViT" (1793) #11 "swin transformer" (3) #12 "detection" (42060) #13 "diagnosis" (221647) #14 "segmentation" (1447) #15 "dental radiograph" (5) #16 "panoramic radiograph" (85) #17 "periapical radiograph" (223) #18 #1 OR #2 (1982) #19 #3 OR #4 OR #5 (7082) #20 #6 OR #7 OR #8 OR #9 OR # 10 OR #12 (1430086) #21 #12 OR #13 OR #14 (248098) #22 #15 OR #16 OR #17 (310) #23 #18 AND #19 AND #20 AND #21 AND #22 (0)	0
Embase	('alveolar bone loss'/exp OR 'alveolar bone loss' OR 'alveolar resorption' OR 'mandible alveolar bone loss' OR 'periodontal bone loss') AND ('deep learning'/exp OR 'deep ml' OR 'deep learning' OR 'deep machine learning' OR 'hierarchical learning' OR 'transformer model'/exp) AND ('convolutional neural network'/exp OR 'cnn (convolutional neural network)' OR 'cnns (convolutional neural networks)' OR 'convnet' OR 'convoluted neural network' OR 'convolution neural network' OR 'convolutional anns' OR 'convolutional nn' OR 'convolutional artificial neural network' OR 'convolutional deep neural network' OR 'convolutional neural network' OR 'convolutional neural networks' OR 'convolutionary neural network' OR 'deep convolutional neural network') AND ('diagnostic procedure'/exp OR 'diagnosis, measurement and analysis' OR 'diagnostic method' OR 'diagnostic procedure' OR 'diagnostic procedures' OR 'diagnostic techniques' OR 'diagnostic techniques and procedures' OR 'diagnostic accuracy'/exp OR 'accuracy, diagnostic' OR 'diagnosis accuracy' OR 'diagnostic accuracy' OR 'diagnostic test accuracy')	23
Worldcat	kw: Periodontal bone loss AND kw: deep learning AND kw: cnn AND kw: detection AND kw: dental radiograph	46
Lens	("periodontal bone loss" OR "alveolar bone loss") AND ("deep learning" OR "artificial intelligence" OR "machine learning") AND ("convolutional neural network" OR "CNN" OR "transformer" OR "vision transformer" OR "ViT" OR "Swin Transformer") AND ("detection" OR "diagnosis" OR "segmentation") AND ("dental radiograph" OR "panoramic radiograph" OR "periapical radiograph")	93
Google Scholar	"periodontal bone loss" AND ("deep learning" OR "artificial intelligence" OR "machine learning") AND ("transformer model" OR "convolutional neural network" OR "vision transformer" OR "Swin Transformer") AND ("detection" OR "diagnosis") AND ("accuracy" OR "diagnosis") AND "dental radiograph"	69
Taylor & Francis	("periodontal bone loss" OR "alveolar bone loss") AND ("convolutional neural network" OR "CNN" OR "transformer" OR "vision transformer" OR "ViT" OR "Swin Transformer") AND ("detection" OR "diagnosis" OR "segmentation")	6
Science Direct	("periodontal bone loss" OR ―alveolar bone loss‖) AND ("deep learning" OR "artificial intelligence") AND ("convolutional neural network" OR "transformer") AND (detection OR diagnosis) AND "dental radiograph"	40
ClinicalTrials.gov	Condition/disease: Periodontal Bone Loss OR Alveolar Bone Loss Other Terms: - Intervention/Treatment: convolutional neural network OR CNN based model OR Vision Transformer Location:-	0

###  Eligibility Criteria

 Studies were eligible for inclusion if they met the following criteria: (1) original research articles (experimental, retrospective, prospective, or cross-sectional) using deep learning models; (2) studies involving adult participants (≥ 18 years) with anterior and/or posterior dental radiographs; (3) studies that diagnosed PBL, either by detecting its presence or classifying its severity; (4) studies that employed deep learning models based on CNN and/or transformer architectures (e.g., ViT, BEiT, Swin, DeiT); (5) studies that used radiographic data (periapical, bitewing, or other dental radiographs) from real human subjects; (6) studies that reported at least one quantitative diagnostic performance metric, such as AUC, sensitivity, specificity, F1-score, precision/recall, correlation (PCC/ICC), or overall diagnostic accuracy; and (7) articles published in English between January 2015 and June 2025. The exclusion criteria were as follows: (1) review articles, protocols, conference abstracts, editorials, commentaries, and case reports; (2) studies involving animals, simulations, phantom data, or pediatric populations (< 18 years); (3) studies that did not evaluate PBL but focused on other dental conditions (e.g., caries, abscesses); (4) studies that used non-deep learning AI methods (e.g., SVM, decision trees, random forests); and (5) studies that did not report relevant diagnostic performance metrics or only provided qualitative or subjective evaluations.

###  Data Extraction

 Data extraction was performed independently by two authors using a standardized extraction form. Extracted data included: (1) study identification details (first author, year of publication); (2) study design and population characteristics (radiograph type, sample size, patient condition); (3) type of artificial intelligence model used (e.g., CNN, transformer, or hybrid), including specific architectures; (4) task performed (e.g., detection, classification, segmentation); and (5) primary outcome measures related to model performance, such as accuracy, AUC, sensitivity, specificity, and F1-score. Information related to dataset size, validation methods, and reference standards was also collected where available.

###  Risk of Bias Assessment

 The risk of bias and methodological quality of the included studies were assessed independently by all authors using the updated Prediction model Risk Of Bias Assessment Tool for Artificial Intelligence (PROBAST+AI). This tool is specifically tailored for evaluating machine learning and deep learning-based prediction models in healthcare, including diagnostic applications involving medical imaging. It consists of 18 signaling questions organized into four key domains: Participants, Predictors, Outcome, and Analysis. Each domain was critically evaluated for potential bias and rated as low, high, or unclear risk of bias according to standardized guidance provided in the PROBAST+AI manual. This tool also incorporates AI-specific considerations, such as the transparency of ground truth labeling, strategies to mitigate overfitting, handling of training/validation/test splits, and model interpretability methods. In instances of discrepancy between assessors, disagreements were resolved through discussion, and where necessary, final judgments were determined by majority consensus.^15^

###  Data Synthesis and Statistical Methods

 Meta-analysis was conducted using RStudio (version 2024.12.1+563, R Foundation for Statistical Computing, Vienna, Austria) utilizing the meta package to evaluate the diagnostic performance of artificial intelligence (AI) models in detecting PBL. A pairwise meta-analysis approach was applied to compare the pooled diagnostic accuracy of CNN and transformer-based models. Prior to analysis, studies were categorized into two primary groups based on their fundamental feature extraction architecture: CNN and transformer. The CNN group comprised purely CNN-based studies alongside hybrid CNN studies. These hybrid models, which employed CNN backbones such as DenseNet121 or AlexNet combined with classical classifiers (e.g., SVM) or supplementary algorithmic layers, were grouped into the CNN category because their primary image representation and feature extraction are performed by convolutional layers. Similarly, the transformer group included purely transformer-based architectures and one hybrid transformer study (SegFormer), which was grouped into this category because its core mechanism relies on a transformer encoder to capture global spatial dependencies, the defining characteristic of its diagnostic approach. Effect estimates were generated separately for each group.

 The primary outcome was the Diagnostic Odds Ratio (DOR), calculated from two-by-two contingency tables consisting of true positives (TP), false negatives (FN), false positives (FP), and true negatives (TN). Only studies reporting binary classification results (i.e., healthy vs. periodontitis) were included. Pooled DORs were estimated using a random-effects model with the DerSimonian-Laird (DL) estimator to account for inter-study heterogeneity. Study weights were assigned using the inverse-variance method, and a continuity correction of 0.5 was applied to zero-cell counts. Forest plots were generated to display individual and pooled estimates with corresponding 95% confidence intervals (CIs). Heterogeneity was evaluated using Cochrane’s Q test and I^2^ statistic, with values of I^2^ > 50% and *P* < 0.05 considered substantial. To explore sources of heterogeneity, a subgroup analysis was conducted based on model type. Furthermore, to assess the robustness of the findings, a leave-one-out sensitivity analysis was performed using the metainf function, whereby each study was sequentially omitted to evaluate its influence on the overall pooled effect. All statistical tests were two-sided, and *P*-values < 0.05 were considered statistically significant.

## Results

###  Selection of Studies

 A total of 347 records were identified from ten databases. After removing 109 duplicates, 238 records remained. Screening of titles and abstracts excluded 165 records. Of the 73 full texts retrieved, 2 could not be obtained. The remaining 71 full-text articles were assessed, and 48 were excluded for the following reasons: editorials (n = 1), letters (n = 1), theses (n = 2), conference abstracts (n = 6), reviews (n = 15), non-English articles (n = 1), and irrelevant variables (n = 22). Of the 23 studies that met the inclusion criteria, a risk-of-bias assessment identified 6 studies with a high risk of bias. This resulted in 17 final included studies (Figure 1).

**Figure 1:**
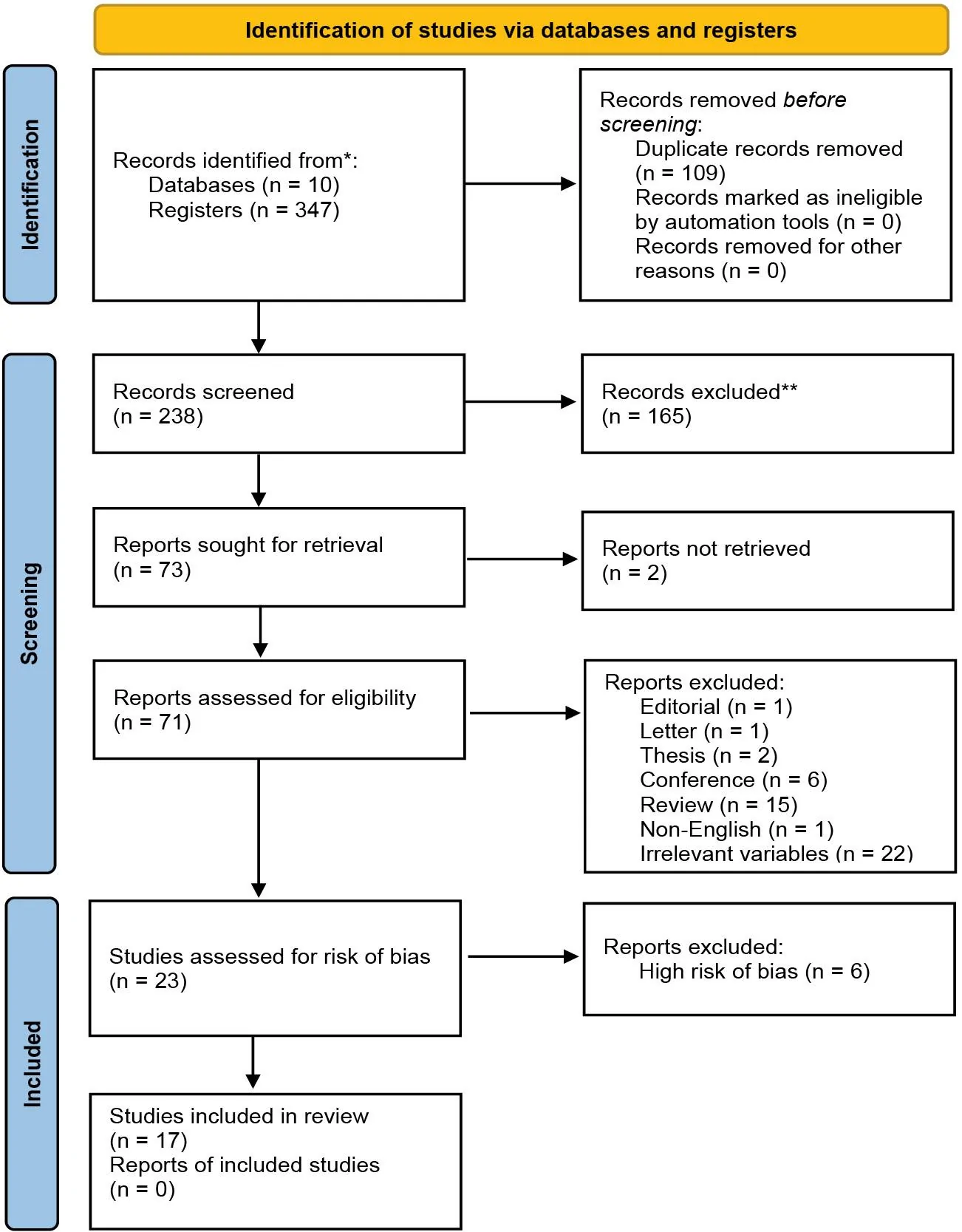
PRISMA 2020 flow diagram of the screening protocol

###  Risk of Bias

 Risk of bias was assessed using the PROBAST+AI tool. Fifteen studies (65.2%) had a low overall risk of bias, six studies (26.1%) had a high risk, and two studies (8.7%) had unclear risk. High-risk ratings were primarily in the Outcome and Analysis domains. Figure 2 shows domain-level ratings.

**Figure 2:**
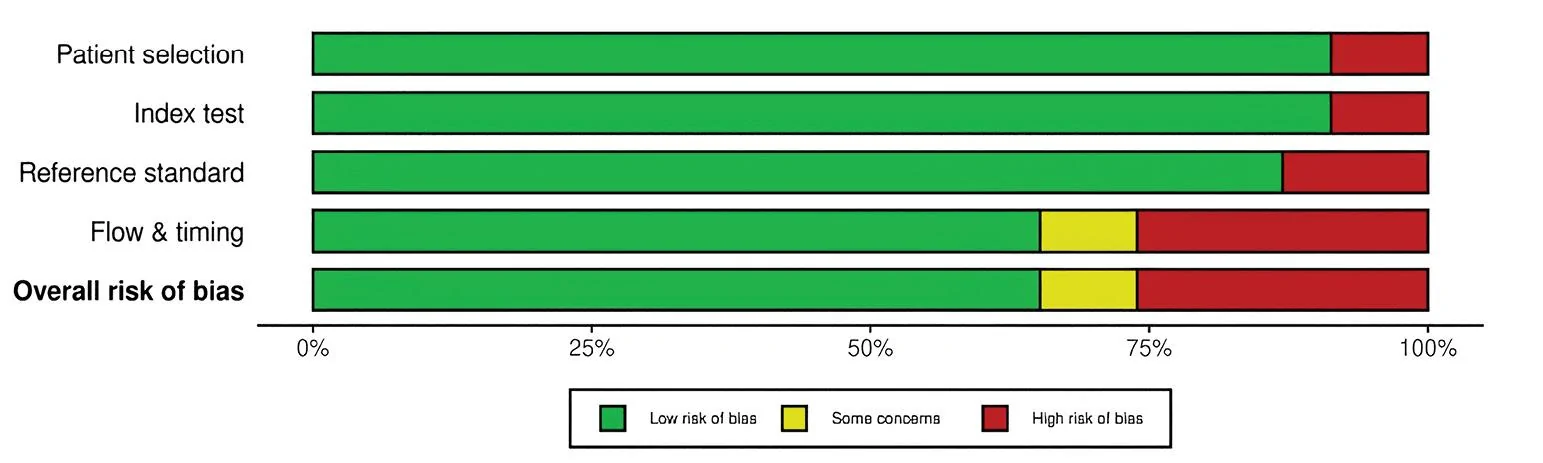
Quality assessment of included articles presented using summary plots

###  Characteristics of Studies

 Seventeen studies met the criteria for inclusion in the meta-analysis, comprising 14 studies employing CNN-based or hybrid CNN architectures (Study No. 1–14) and three studies utilizing transformer-based or hybrid transformer architectures (Study No. 15–17), with publication years ranging from 2019 to 2025. Panoramic radiographs were the most frequently used imaging modality (n = 7), followed by periapical radiographs alone (n = 4), periapical combined with bitewing (n = 2), bitewing alone (n = 2), CBCT (n = 1), and mixed or unspecified intraoral radiographs (n = 1). The pooled dataset size for CNN-based and hybrid CNN studies was 22,914 event (bone loss) cases and 53,529 control (healthy) cases, whereas transformer-based and hybrid transformer studies analyzed 3,588 event cases and 1,547 control cases. Supplementary File presents the detailed characteristics of each study.

###  Diagnosis Value of CNN and Transformer-based AI Models for PBL

 The pooled sensitivity for CNN-based models, calculated from the included binary classification studies, was 0.85 (95% CI: 0.77–0.90) based on the random-effects model. A high degree of heterogeneity was observed (I^2^ = 96.5%, τ^2^ = 0.6361, *P* < 0.0001), and the prediction interval ranged from 0.45 to 0.97. For transformer-based models, the pooled sensitivity was slightly higher at 0.87 (95% CI: 0.74– 0.94), also with substantial heterogeneity (I^2^ = 98.6%, τ^2^ = 0.5365, *P* < 0.0001) and a prediction interval ranging from 0.14 to 1.00. Figures 3 and 4 present forest plots summarizing individual and pooled sensitivity values for each model type.

**Figure 3:**
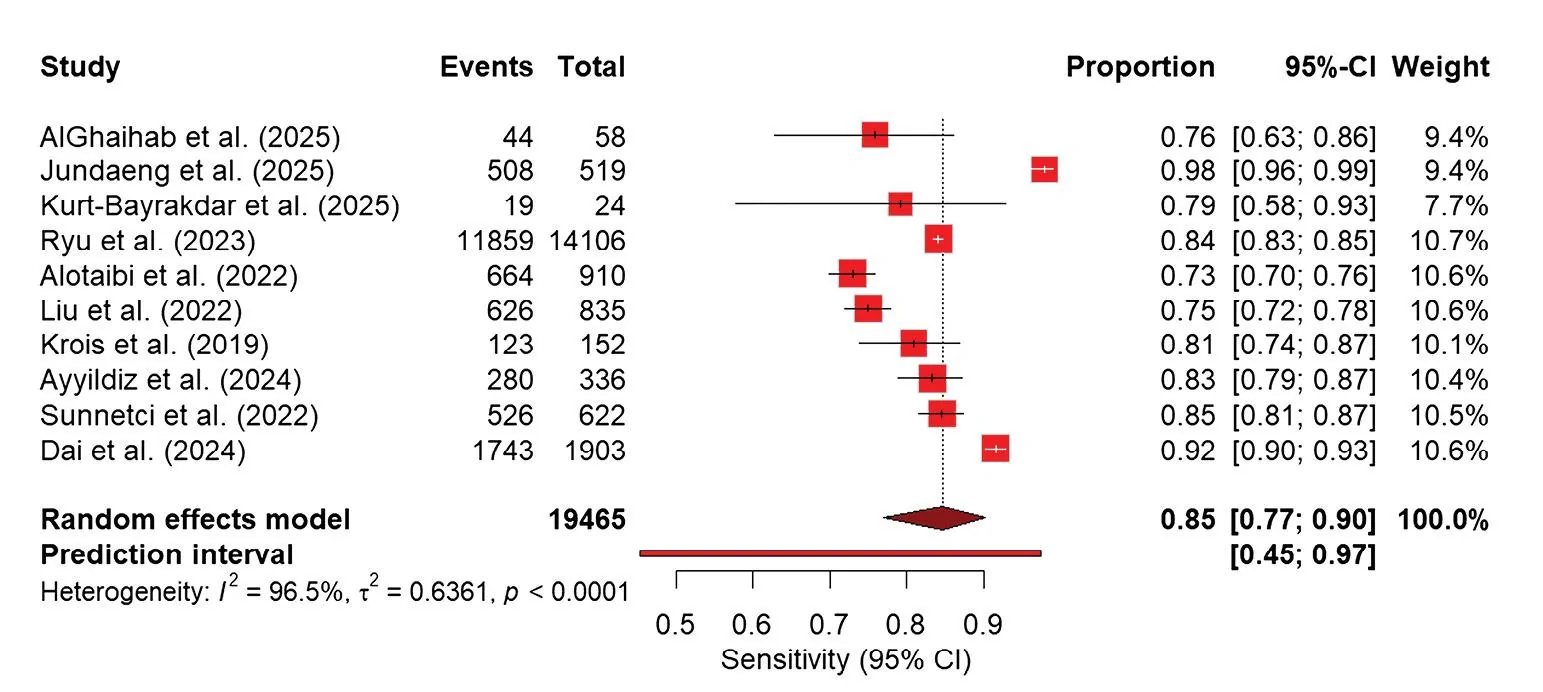
Forest plot of sensitivity estimates for CNN-based AI models in the detection of PBL^10-12,16,18,19,21-23,26^

**Figure 4:**
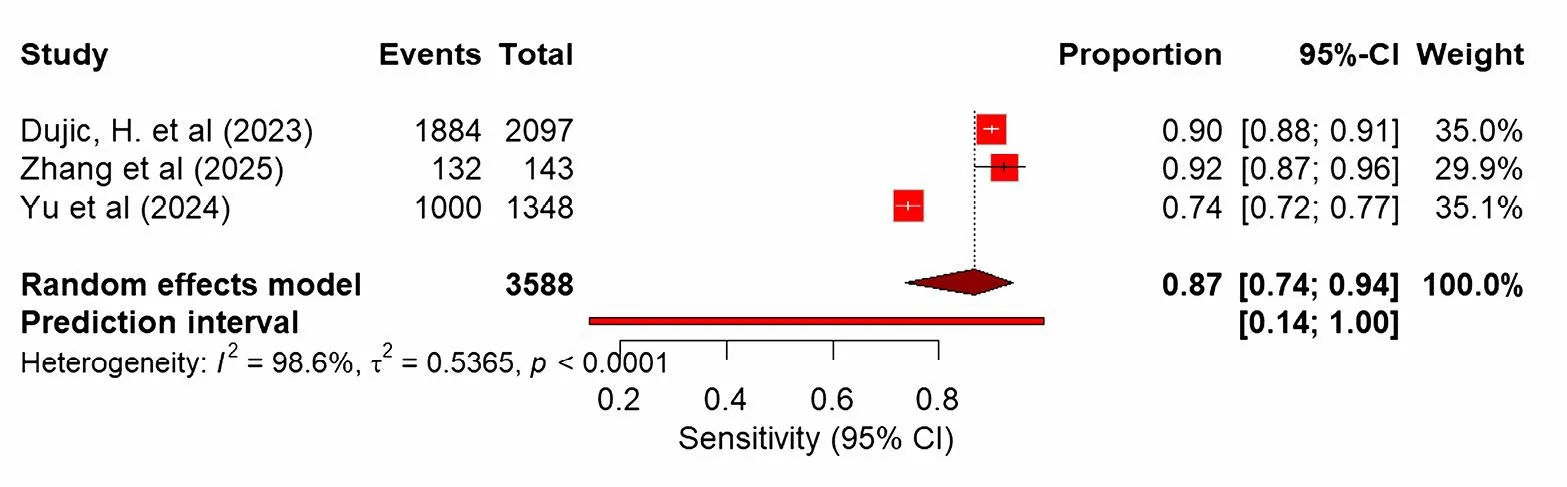
Forest plot of sensitivity estimates for transformer-based AI models in the detection of PBL^7,13,27^

 The pooled specificity for CNN-based models, derived from the same set of eligible studies, was 0.88 (95% CI: 0.80–0.93) using the random-effects model. Heterogeneity was considerable (I^2^ = 99.4%, τ^2^ = 1.0152, *P* < 0.0001), with a prediction interval ranging from 0.40 to 0.99. Transformer-based models had a pooled specificity of 0.80 (95% CI: 0.34–0.97), with similarly high heterogeneity (I^2^ = 98.5%, τ^2^ = 3.0921, *P* < 0.0001) and a broader prediction interval from 0.00 to 1.00. Figures 5 and 6 present forest plots for the specificity analysis.

**Figure 5:**
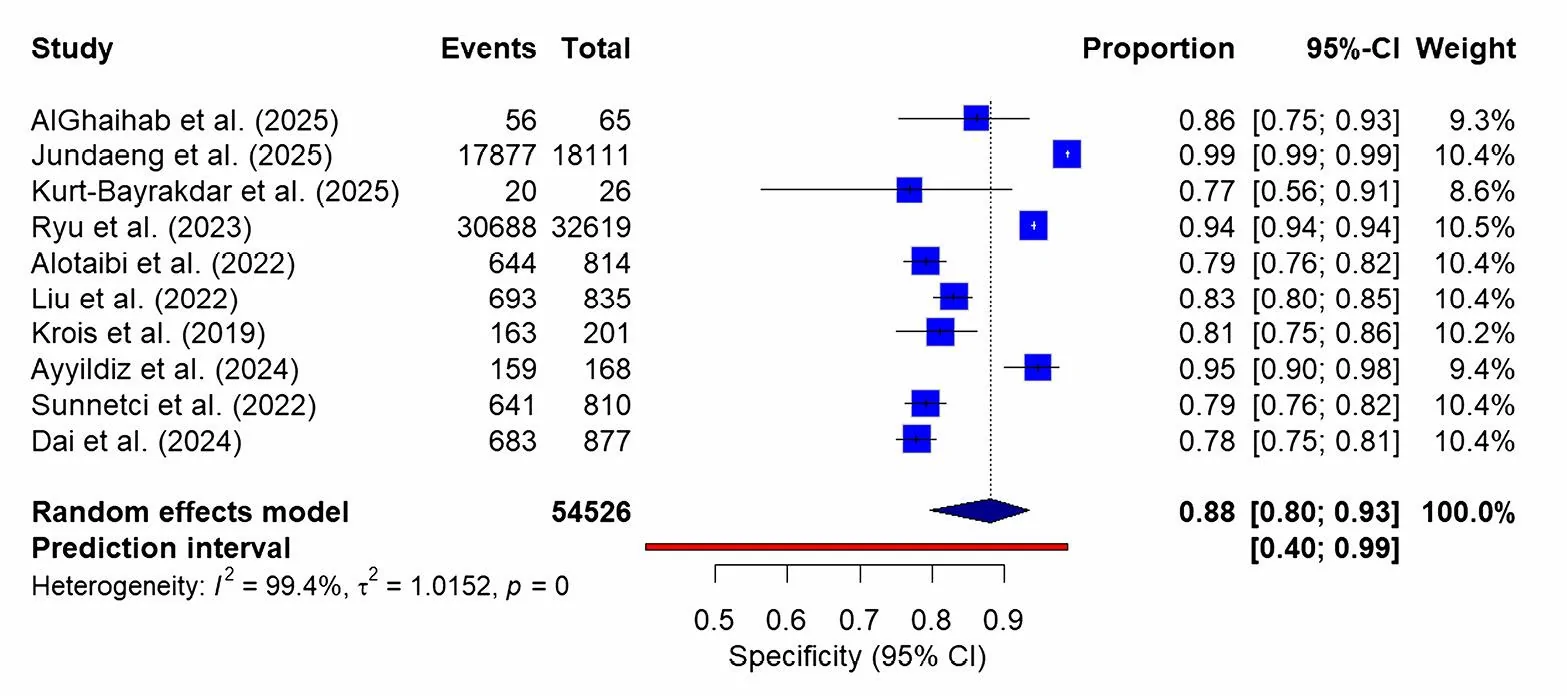
Forest plot of specificity estimates for CNN-based AI models in the detection of PBL^10-12,16,18,19,21-23,26^

**Figure 6:**
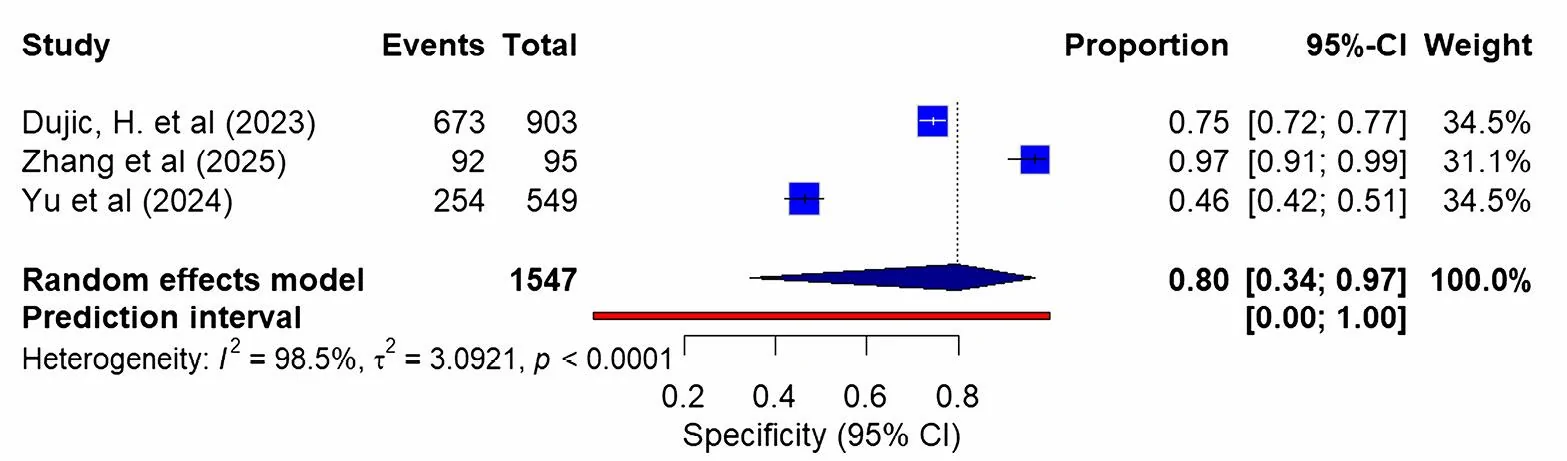
Forest plot of specificity estimates for transformer-based AI models in the detection of PBL^7,13,27^

 For diagnostic odds ratio (DOR), the pooled estimate for CNN-based models was 41.69 (95% CI: 19.41–89.52; *P* < 0.0001) under the random-effects model. Heterogeneity was high (I^2^ = 98.8%, τ^2^ = 1.4248), indicating substantial variation in effect sizes across studies. For transformer-based models, the pooled DOR was 25.64 (95% CI: 3.47–189.63; *P* < 0.0001), with a heterogeneity index of I^2^ = 99.3% (τ^2^ = 2.9847). Forest plots displaying the individual study estimates and pooled DOR values for each architecture are shown in Figures 7 and 8.

**Figure 7:**
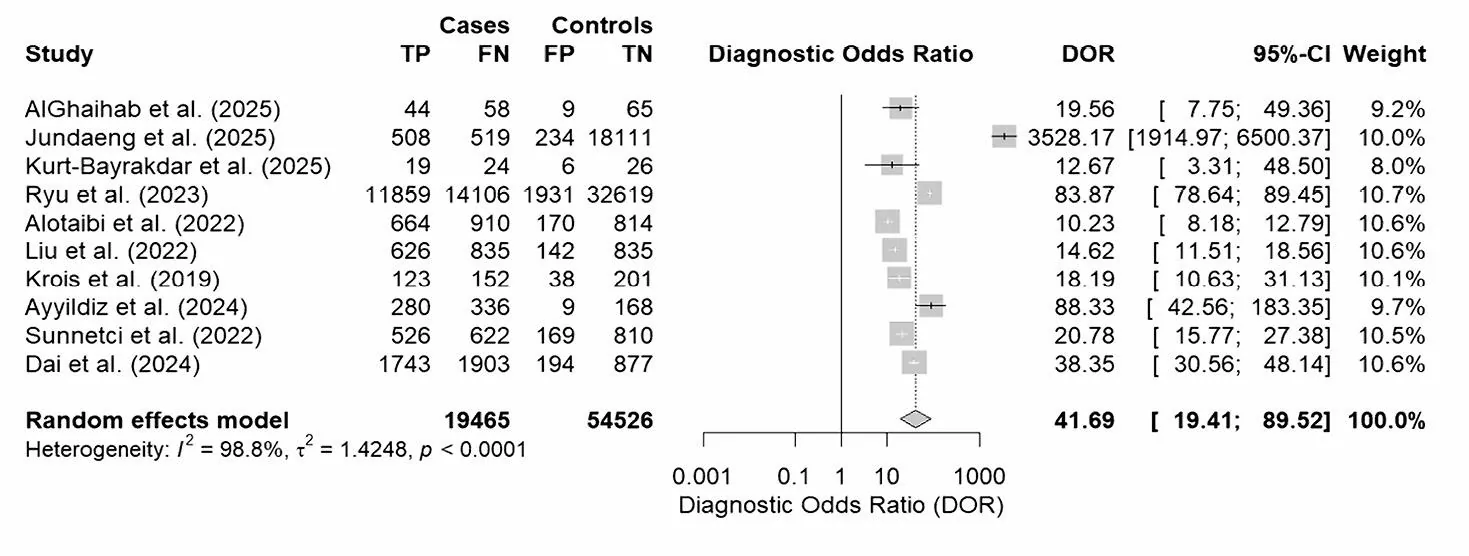
Forest plot of the DOR for CNN-based AI models in the detection of PBL^10-12,16,18,19,21-23,26^

**Figure 8:**
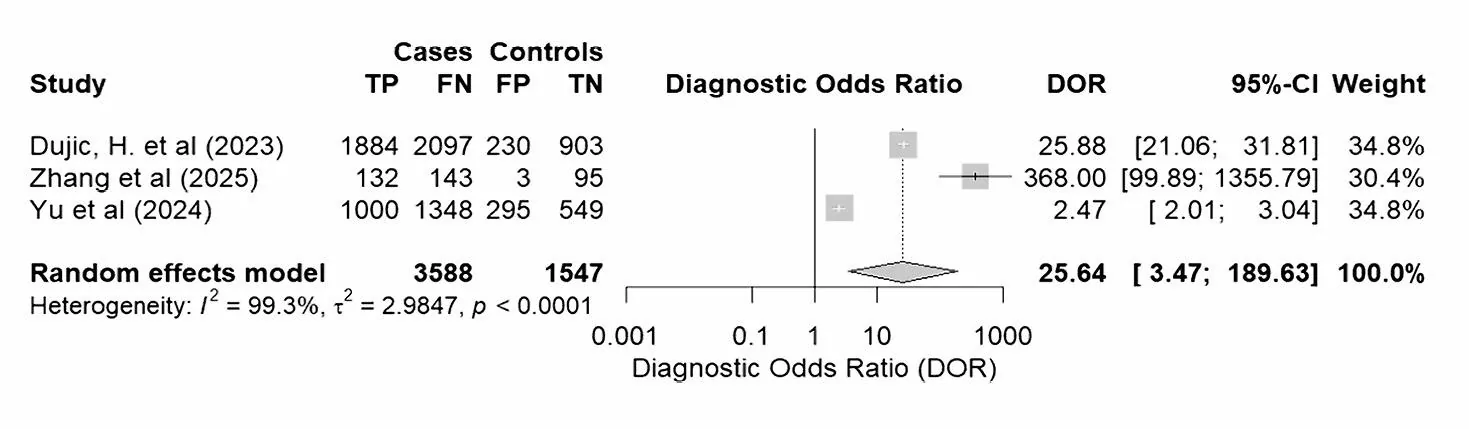
Forest plot of the DOR for transformer-based AI models in the detection of PBL^7,13,27^

###  Heterogeneity and Sensitivity Analysis

 Subgroup analysis for CNN-based models was conducted to explore potential sources of inter-study variability in diagnostic performance. Among the examined factors, radiograph type (*P* = 0.0309) and study design (*P* = 0.0468) showed statistically significant contributions to heterogeneity. Studies using panoramic radiographs and those with prospective or unclear study designs yielded higher pooled DOR values than other categories. In contrast, CNN architecture (*P* = 0.8729), geographical setting (*P* = 0.1320), and dataset size (*P* = 0.1811) did not demonstrate statistically significant differences in subgroup comparisons. Figure 9 illustrates the complete subgroup analysis results for radiograph type in CNN-based models.

**Figure 9:**
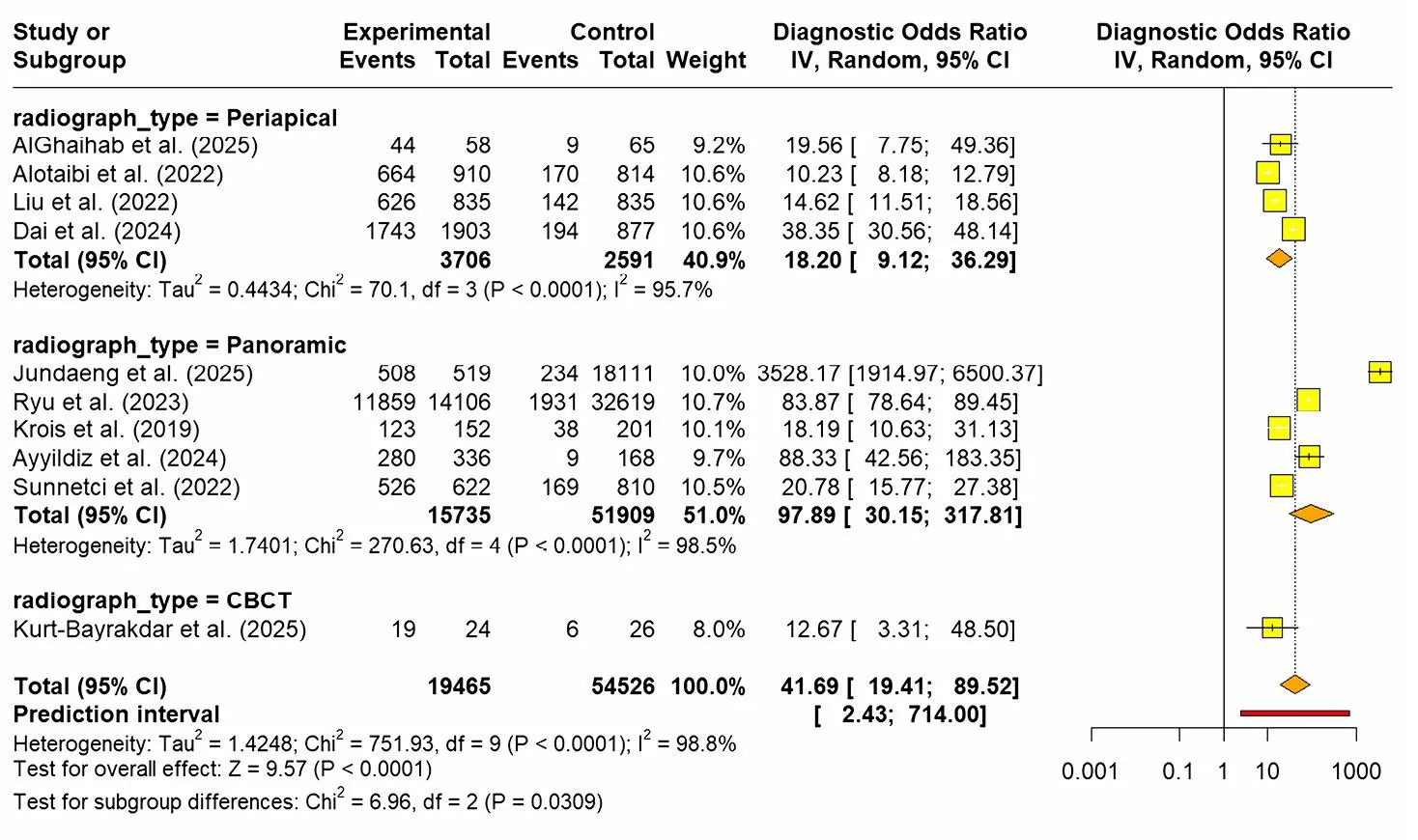
Subgroup analysis of DOR by radiograph type^10-12,16,18,19,21-23,26^

 Leave-one-out sensitivity analysis showed that CNN-based pooled DOR values ranged from 25.54 to 49.36, all statistically significant (*P* < 0.0001). In the transformer group, excluding Zhang et al.^7^ (2025) reduced the pooled DOR to 8.00 (95% CI: 0.80–79.87; *P* = 0.0764), while excluding other studies yielded values between 28.90 and 89.97 (Figures 10 and 11).

**Figure 10:**
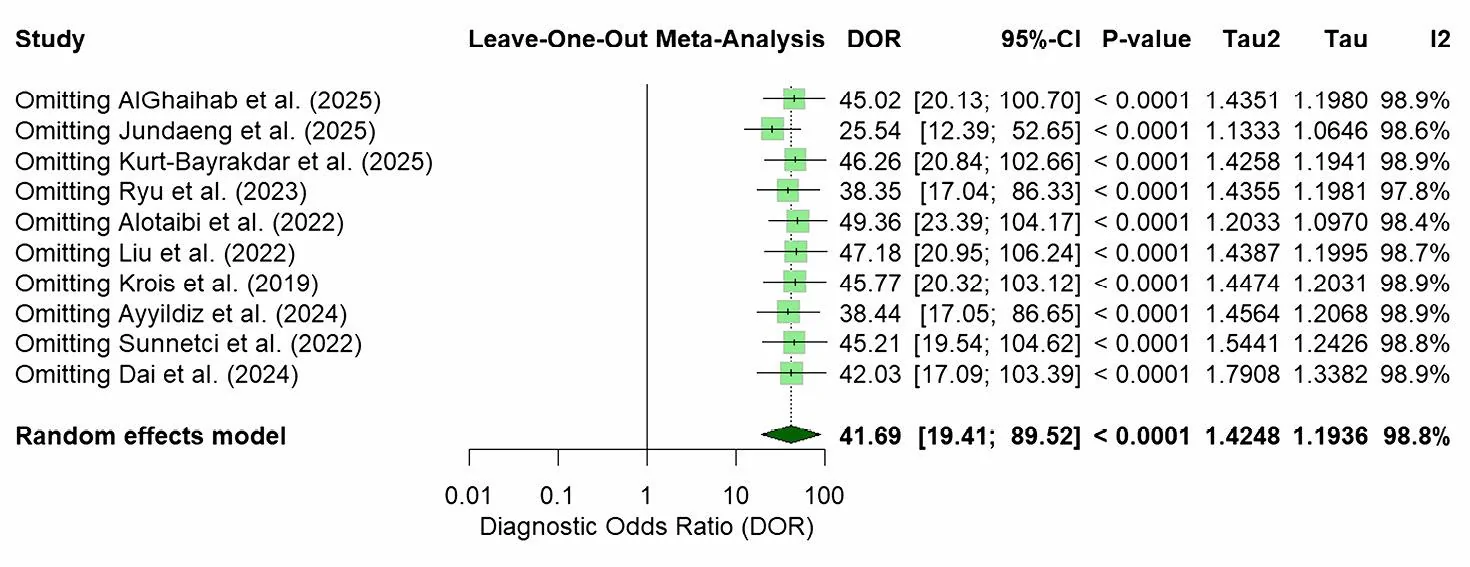
Leave-one-out sensitivity analysis of pooled odds ratio for CNN-based AI models in detecting PBL^10-12,16,18,19,21-23,26^

**Figure 11:**
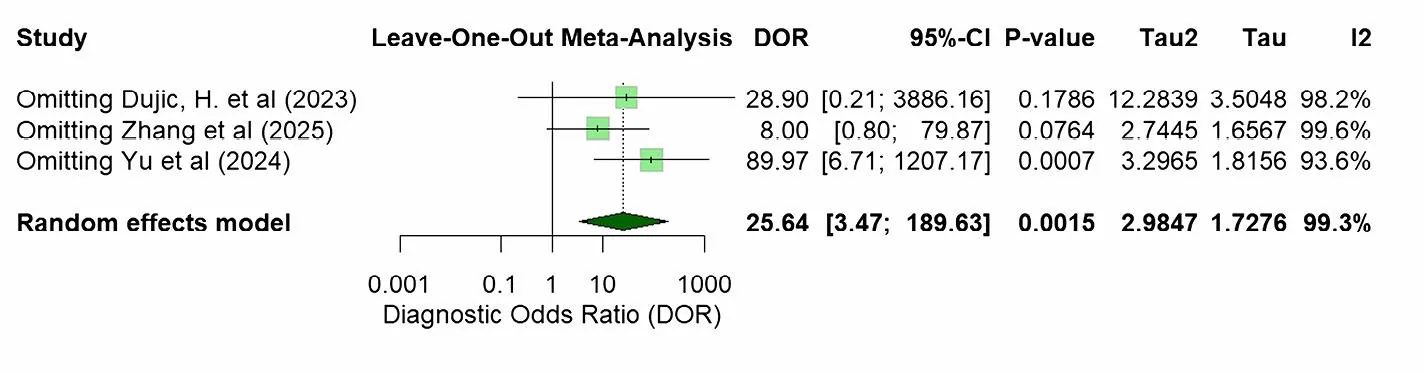
Leave-one-out sensitivity analysis of pooled odds ratio for transformer-based AI models in detecting PBL^7,13,27^

## Discussion

 According to Supplementary File, the highest diagnostic accuracy for PBL detection was achieved by the CNN model (0.98), largely due to the accuracy metric’s dependence on the proportion of TP and TN. Leveraging its local feature extraction approach, the CNN was more selective in classifying positive PBL cases, leading to a higher number of TNs, while still detecting an adequate number of TP cases through preprocessing and augmentation, which enhanced PBL visibility and reduced overfitting.^7,18,21^ In contrast, the ViT in Zhang et al.^7^ (2025) study achieved the highest AUC by effectively ranking positive cases above negative ones across all thresholds, maintaining high TP rates with low FP rates through high-quality CBCT-based labels, extensive augmentation, and the ability to capture both global and local image features. This same architecture and training rigor also produced the highest precision, recall, and F1 scores, as it accurately identified true cases while minimizing both FP and FN outcomes.^7^ Meanwhile, the hybrid transformer model in Yu et al.^13^ (2024) study attained the highest correlation (r≈0.85–0.87) between predicted and clinician-measured RBL across 1,898 teeth by combining accurate segmentation, stable PCA-based axis determination, consistent measurement protocols, standardized high-quality images, and a large, well-matched test set.^13^ These are factors that minimized prediction errors and maintained a tightly linear relationship between model outputs and clinical measurements.

 Based on Figures 3 and 4, the highest pooled sensitivity for PBL detection was achieved by the transformer model, at 0.87 (95% CI: 0.74–0.94). This advantage stems from the transformer architecture’s ability to model global context via self-attention, enabling it to identify complex patterns and spatial relationships that CNN-based models often overlook.^7,21^ Based on Figures 5 and 6, the CNN model showed the highest pooled specificity for PBL detection, which was 0.88 (95% CI: 0.80–0.93). This achievement can be attributed to the CNN architecture’s focus on local feature extraction, thereby identifying only visually clear PBL images, but it limits detection in cases of minimal bone loss or complex structures.^7,12,13,17^ This condition leads to an increased tendency for non-PBL case classification, which ultimately contributes to the high specificity value.

 The meta-analysis showed that CNN-based deep learning models (Figure 7) achieved strong diagnostic performance in detecting periodontal bone loss, with a pooled DOR of 41.69 (95% CI: 19.41–89.52, O < 0.0001), while transformer-based models (Figure 8) also demonstrated significant efficacy with a pooled DOR of 25.64 (95% CI: 3.47–189.63). However, extremely high heterogeneity (I^2^ = 98.8% for CNNs and 99.3% for transformers) and wide confidence intervals limit the certainty of these results. Subgroup analyses (Figure 9) identified radiograph type (*P* = 0.0309) and study design (*P* = 0.0468) as key contributors, with panoramic radiographs and prospective or unclear designs showing higher pooled DOR. Variability likely stems from differences in architectures, datasets, labeling, and imaging modalities. Although the ViT by Zhang et al.^7^ (2025) achieved an exceptional DOR of 368.00, small sample sizes and a lack of standardization limit generalizability, highlighting the need for consistent protocols and robust multicenter validation.

 Based on the results (Figures 10 and 11), the CNN-based findings showed no evidence that any single study disproportionately influenced the pooled DOR, indicating stable performance despite consistently high heterogeneity. In contrast, the transformer-based results were highly sensitive to the exclusion of certain studies, most notably Zhang et al.^7^ (2025), which reduced the pooled DOR to a non-significant level. The substantial fluctuations in effect size and heterogeneity metrics across iterations highlight potential instability in transformer model estimates, underscoring the need for cautious interpretation compared with the more robust CNN-based findings.

 This study has several limitations. The meta-analysis showed very high heterogeneity for both CNN (I^2^ = 98.8%) and transformer-based (I^2^ = 99.3%) models, partly due to variations in radiograph types, study designs, dataset sizes, and labeling quality. The limited number of transformer studies (n = 3) made pooled estimates unstable, with results highly sensitive to the exclusion of individual studies. Differences in outcome definitions, preprocessing methods, and evaluation metrics may have affected comparability, while small sample sizes and single-center datasets in some studies may have inflated performance estimates. Excluding studies with non-comparable metrics may also have introduced selection bias.

 Adopting CNN and transformer-based models in dental imaging can improve early detection of periodontal bone loss (PBL), enabling timely, personalized care. Clinical adoption requires validation across diverse datasets, enhanced interpretability, high-quality annotated data, and standardized protocols to ensure reliable, collaborative, and safe use.

## Conclusion

 This systematic review and meta-analysis demonstrated that CNN-based deep learning models achieve high and statistically robust diagnostic accuracy for periodontal bone loss detection, with pooled sensitivity. Transformer-based models demonstrated diagnostic promise; however, estimates from only three studies were characterized by wider confidence intervals, greater inter-study variance, and high sensitivity to the exclusion of individual studies, precluding reliable comparative inference. The data therefore indicate that CNN-based models are currently supported by a larger and more statistically robust body of evidence, though a definitive claim of superiority remains unwarranted. Adequately powered, multicenter comparative studies under standardized conditions are needed before firm architectural conclusions can be drawn.

## Competing Interests

 The authors declare no conflicts of interest.

## Data Availability

 The datasets analyzed during the current study are available from the corresponding author upon reasonable request.

## Ethical Approval

 Not applicable. This study is a systematic review and meta-analysis of previously published data and did not involve direct human subjects. Therefore, ethical approval was not required.

## Supplementary Files

Study Characteristics.
